# Facial Width-To-Height Ratio Relates to Alpha Status and Assertive Personality in Capuchin Monkeys

**DOI:** 10.1371/journal.pone.0093369

**Published:** 2014-04-04

**Authors:** Carmen Emilia Lefevre, Vanessa A. D. Wilson, F. Blake Morton, Sarah F. Brosnan, Annika Paukner, Timothy C. Bates

**Affiliations:** 1 Centre for Decision Research, Leeds University Business School, Leeds, United Kingdom; 2 Department of Psychology, University of Edinburgh, Edinburgh, United Kingdom; 3 Scottish Primate Research Group, Edinburgh, United Kingdom; 4 Behaviour and Evolution Research Group, Psychology, School of Natural Sciences, University of Stirling, Stirling, United Kingdom; 5 Department of Psychology and Language Research Centre, Georgia State University, Atlanta, Georgia, United States of America; 6 Laboratory of Comparative Ethology, Eunice Kennedy Shriver National Institute of Child Health and Human Development, National Institutes of Health, Department of Health and Human Services, Poolesville, Maryland, United States of America; Institut Pluridisciplinaire Hubert Curien, France

## Abstract

Social dominance hierarchies play a pivotal role in shaping the behaviour of many species, and sex differences within these hierarchies often exist. To date, however, few physical markers of dominance have been identified. Such markers would be valuable in terms of understanding the etiology of dominant behaviour and changes in social hierarchies over time. Animals may also use such traits to evaluate the potential dominance of others relative to themselves (i.e. a physical “cue”). Facial width-to-height ratio (fWHR), for example, has been suggested as a cue to dominance in humans, with links to both dominant behaviour and the perception of dominance in other individuals. Whether this association is present in non-human animals is currently not known. Therefore, here we examine within-species links between fWHR and dominant behaviour in 64 brown capuchin monkeys (*Sapajus spp.*) aged between 2 and 40 years. fWHR was positively associated with alpha status and with a dimensional rating of assertive personality in both males and females. Moreover, fWHR showed significant sexual dimorphism in adults but not juveniles, suggesting a developmental change may occur during puberty. In a sub-sample, sex differences were mediated by weight, suggesting fWHR dimorphism does not exceed what would be expected by differences in body weight. This is the first report of an association between face shape and behaviour in a non-human species. Results are discussed in terms of the role that face-behaviour associations might play within capuchin societies, and the possible selective forces that might have led to the evolution of fWHR-dominance associations in humans.

## Introduction

In many species, competitive inter- and intra-group encounters between rivalling individuals are common and typically aggressive (e.g. [1], [2]). Nonetheless, few external physical measures have been identified to date that appear to mediate these behavioural traits across and within species. For instance, species-level differences in canine size are associated with the frequency and costs of contest competition (e.g. [1]), while body size has been linked to social rank in various species, including, for instance, primates (e.g. [3], [4]) and elephant seals [5]. Additional quantifiable physical traits linked to social rank or assertive behaviour would be valuable as these may facilitate a better understanding of the etiology of dominance in animals, including humans. Accordingly, here we report on a candidate cue to dominant behaviour, the facial width-to-height ratio (fWHR), in brown capuchin monkeys (*Sapajus spp.*; hereafter referred to as *Sapajus*; see [6] for recent taxonomy change). Like humans, in which fWHR has been related to dominance behaviours [8]–[12], *Sapajus* exhibit low canine dimorphism and are therefore an ideal non-human primate species in which to test the relationship between fWHR and correlates of dominance. fWHR was first assessed with attention to its sexual dimorphism in a range of primate species including *Sapajus* [7]. Weston et al. [7] reported an association between fWHR and canine size dimorphism, whereby species with large sexual dimorphism in canine size exhibit less sexual dimorphism in fWHR [7]. Importantly, however, Weston et al. [7] only discussed relative size differences between males and females (i.e. sexual dimorphism) within one species, but not overall size or size differences between species for either fWHR or canine height. Following this initial work, a range of studies, thus far conducted exclusively in humans, have found associations between fWHR and behaviours related to the acquisition of social status. For example, in human males higher fWHR is associated with deception [8], achievement striving [9], decreased rates of reciprocation in economic games [10], increased rates of self-sacrifice for the in-group [11], and, of particular interest here, elevated aggression [12], although the size and robustness of the latter effect is somewhat unclear [13], [14]. In addition, several studies of humans have shown that fWHR is related to the perceived dominance and dominance-linked behaviours of others, suggesting that fWHR may serve as a physical cue to one’s status within a group [10], [15], [16]. It is currently unclear, however, whether fWHR is linked to behaviours associated with dominance linked traits in other animals. Here, in order to address this question, we test whether fWHR in *Sapajus* is associated with alpha status and a dimensional rating of assertive personality (hereafter “Assertiveness”; Morton et al. 2013). Testing the link between fWHR and status/Assertiveness in a nonhuman primate species may help with understanding the biological and evolutionary bases of fWHR-dominance relationships in humans.

### Sexual Dimorphism in fWHR

Masterson [17] reported consistent sex differences in bizygomatic breadth in adult, but not juvenile, *Sapajus.* Also, as noted above, Weston et al. [7] reported a reciprocal relationship between dimorphism in fWHR and canine size across primate species. *Sapajus*, while having relatively large canines, show little sexual dimorphism in this trait and therefore would be predicted to show significant dimorphism in fWHR, as was found by Weston et al. [7]. However, there are outliers to this trend. For instance, while initial reports indicated that fWHR was dimorphic in humans [12], [18], larger studies suggest a lack of dimorphism for this trait (e.g. [14], [19], [20]). Thus, humans lack significant dimorphism in fWHR and show minimal dimorphism in canine size [21], suggesting that canine size dimorphism may not fully account for species differences in fWHR dimorphism.

Our hypotheses with respect to dimorphism were as follows: Firstly, both studies that previously assessed sex differences in facial width in *Sapajus* [7], [17] measured fWHR from the skull. However, these measures may not be informative with respect to the signalling power of fWHR if they do not translate to the skin surface. Therefore, we wished to replicate findings for *Sapajus* fWHR using measurements taken from the skin surface. These incorporate not only skull, but also muscle and soft tissue differences affecting fWHR, thereby reflecting the visible phenotype of fWHR. Additionally, theory concerning dominance cues in humans suggests a link between dominant behaviour and testosterone (e.g. [22]), with a pubertal spike in testosterone and consequent changes in morphology and behaviour [23], [24]. Such developmental changes may also occur for human fWHR given its association with adult levels of testosterone [25]. We therefore hypothesized, that this skin-surface measure of fWHR would also be sexually dimorphic in *Sapajus*, with males having higher fWHR than females, as reported by Weston et al. [7] for skull measures. Secondly, based on testosterone effects in puberty and in line with findings by Masterson [17], we hypothesised that sex differences in *Sapajus* fWHR would exist among sexually mature, but not sexually immature, individuals.

### fWHR and Dominant Behaviour in Sapajus

*Sapajus* live in relatively small female-bonded arboreal groups [26], [27] that typically include multiple male members [28] as well as both a dominant alpha male and an alpha female [29], with the alpha male being higher-ranking than the alpha female. Cross-species analyses of primates, including capuchins, suggest that such social conditions contribute to lower rates of agonism among conspecifics, and favour facial displays over contact aggression [30]. Indeed, dominance hierarchies in *Sapajus* are, in general, less clearly defined than in Old World primates (e.g. baboons [*Papio spp.*] and rhesus macaques [*Macaca mulatta*]; [31], [32]) and at least among captive capuchin groups, it is difficult to place individuals into discrete dominance ranks given their relatively low rates of aggression and high levels of social tolerance compared to other primate species [32]. Although the alpha male and alpha female are normally easy to identify within *Sapajus* groups, the exact ranking of subordinates is usually less certain, with some studies reporting clear linear hierarchies among *Sapajus*, while others do not [26]. This indicates that *Sapajus* are relatively tolerant of having others in close proximity, and thus may live in more flexible societies compared to many other primate species. Taken together with findings in humans, who (like *Sapajus*) are low on canine dimorphism [21], fWHR may reflect individual differences in dominant behaviour in *Sapajus*, and may even substitute for canine size as a physical cue to one’s capacity for being more (or less) dominant over other individuals. Thus, we predicted that fWHR is associated with alpha status and Assertiveness in this species. Moreover, based on human studies [8], [12] and given that *Sapajus* live in multi-male groups and have flexible dominance hierarchies (factors predicted by Weston et al. [7] to favour reduced canine dimorphism and increased fWHR), we predicted that associations between fWHR and alpha status/Assertiveness in *Sapajus* would hold for both males and females. Lastly, while human fWHR is relatively independent of height and weight [18], several studies indicate that controlling for such body size differences can potentially create artificial links between fWHR and dominant behaviour [20], [34], [33]. We therefore predicted that overall body size partially mediates the relationship between fWHR and alpha status/Assertiveness in *Sapajus*.

## Method

### Ethics Statement

This study was non-invasive, and was approved by the local ethics committees from each research site (Animal Care and Use Committee, NICHD; the Research Committee at Living Links, the Animal Care and Use Committee, GSU), and the Psychology Ethics Committee of the University of Stirling. The study was carried out in strict accordance with the recommendations of the “Guidelines for the treatment of animals in behavioural research and teaching” given by the Association for the Study of Animal Behaviour [35], and the NC3R’s Guidelines for “Primate accommodation, care and use” [36].

### Sample

The sample consisted of a total of 64 individuals (29 female, mean age 12.9 SD = 10.1 years; 35 male, mean age 9.1 SD = 8.6 years) stemming from 7 social groups and a further 4 pair-housings across three sites: The ‘Living Links to Human Evolution’ Research Centre [34] of the University of St Andrews, in Edinburgh Zoo (6 female, mean age 8.2±4.0 years; 10 male, mean age 11.4±13.4 years), the Language Research Center, Georgia State University (13 female, mean age 15.3±11.8 years; 9 male, mean age 10.9±5.80 years), and the Laboratory of Comparative Ethology at the National Institutes of Health, Poolesville, Maryland (10 female, mean age 12.8±9.20 years; 16 male, mean age 6.6±4.50 years). Infants less than one year old were excluded and age was scored by year of life. The sample was additionally categorised according to whether individuals were adult or juvenile. Following [26], adulthood was defined using the criterion of age ≥6 years yielding a sample of 43 adults (with 21 animals classified as juveniles). For a subset of the US individuals, body weight information was available (N = 46, 34 adult). Therefore, we could test for interactions between weight and fWHR among these individuals.

### Site Descriptions

#### Living links, edinburgh zoo

Sixteen capuchins were from the ‘Living Links to Human Evolution’ Research Centre at the Royal Zoological Society of Scotland, Edinburgh Zoo, UK [37]. These individuals were from two breeding groups, and each cohabited with a group of common squirrel monkeys (*Saimiri sciureus*). At the time of this study, the ‘East’ group ranged from 2–3 adult males, 3 adult females, 3 juveniles, and 0–5 infants. The ‘West’ group ranged from 2 adult males, 3 adult females, 4–5 juveniles, and 2–5 infants. All monkeys were captive born except the two eldest males, which were likely wild-born and came to Living Links as established members of the groups. One individual was hand-reared. Both groups were housed in identically designed, but mutually exclusive, 189 m^3^ indoor enclosures with natural light and near-permanent access to a ∼900 m^2^ outdoor enclosure containing trees, providing ample opportunity to engage in natural behaviors. All subjects received commercial TrioMunch pellets supplemented with fresh fruits and vegetables three times daily, and were given cooked chicken and hardboiled eggs once every week. Water was available to the monkeys *ad libitum* at all times and all individuals had full access to proper veterinary care when needed. Further details of housing and husbandry are provided in Leonardi et al. [38].

#### Language research center, georgia state university

Twenty-two capuchins came from three groups at the Language Research Center of Georgia State University (GSU) in Atlanta, Georgia, USA. The first group consisted of 2 adult males, 2 adult females, 2 juveniles, and 0 infants. The second group consisted of 1 adult male, 2 sub-adult males, 2 adult females, 1 juvenile, and 0 infants and the third group consisted of 2 adult males, 8 adult females, and 0 juveniles or infants. All monkeys were captive born. For all groups, enclosures consisted of an indoor room (first group: 75.84 m^3^; second group: 54.42 m^3^, third group: 13.28 m^2^) connected to a large outdoor enclosure (first group: 13.51 m^2^; second group: 21.15 m^2^, third group: 55.74 m^2^). Group members spent most of their time in the outdoor area throughout the year, except when engaged in research, during bad weather, or overnight. Monkeys were provided commercial monkey chow three times a day (morning, noon, evening), and fruits and vegetables were given every evening. Water was available *ad libitum* at all times, including during cognitive and behavioral testing and all individuals had full access to proper veterinary care when needed. The enclosures were made of chain link fencing and were equipped with swings, ropes, and other materials to create three-dimensional living conditions to enrich the monkeys. The older study subjects and third housing group had previously been housed together in various combinations at Yerkes National Primate Research Center, before being relocated to GSU 5 years and 1 year ago respectively.

#### Laboratory of comparative ethology, national institutes of health

Twenty-six capuchins came from two captive breeding group and several small bachelor groups at the Laboratory of Comparative Ethology, NICHD. At the time of the study, one group (Garth’s group) comprised 1 adult male, 4 adult female and 4 juveniles (2 female and 2 male). Three infants (1 female and 2 male, aged <6 months) were part of the group but were not rated for the current study. The second breeding group (Manuel’s group) comprised 1 adult male, 2 adult females, and 4 juveniles (1 female and 3 male). A further nine animals were pair-housed in cages; two pairs and a group of 3 animals were sub-adult to adult males, and one pair was an adult female with a juvenile male. All monkeys were captive born, mother-reared, and housed in the LCE primate facilities at the NIH Animal Center near Poolesville, MD. Breeding groups were housed in one or two parts of three indoor runs (6.9×4.1×2.1 m each) which were connected via sliding doors. Runs were furnished with swings, ladders and various platforms. Cage-housed monkeys were housed in quad cages (1.63×1.63×.71 m per pair). All monkeys were provided with a variety of plastic and metal manipulanda. Monkeys were not food deprived for this study, and received daily nutritional supplements of seeds and fresh fruit or nuts. Commercial monkey biscuits (Labdiet 5045) and water were available *ad libitum* and all individuals had full access to proper veterinary care when needed.

### fWHR Measures

**Figure 1:**
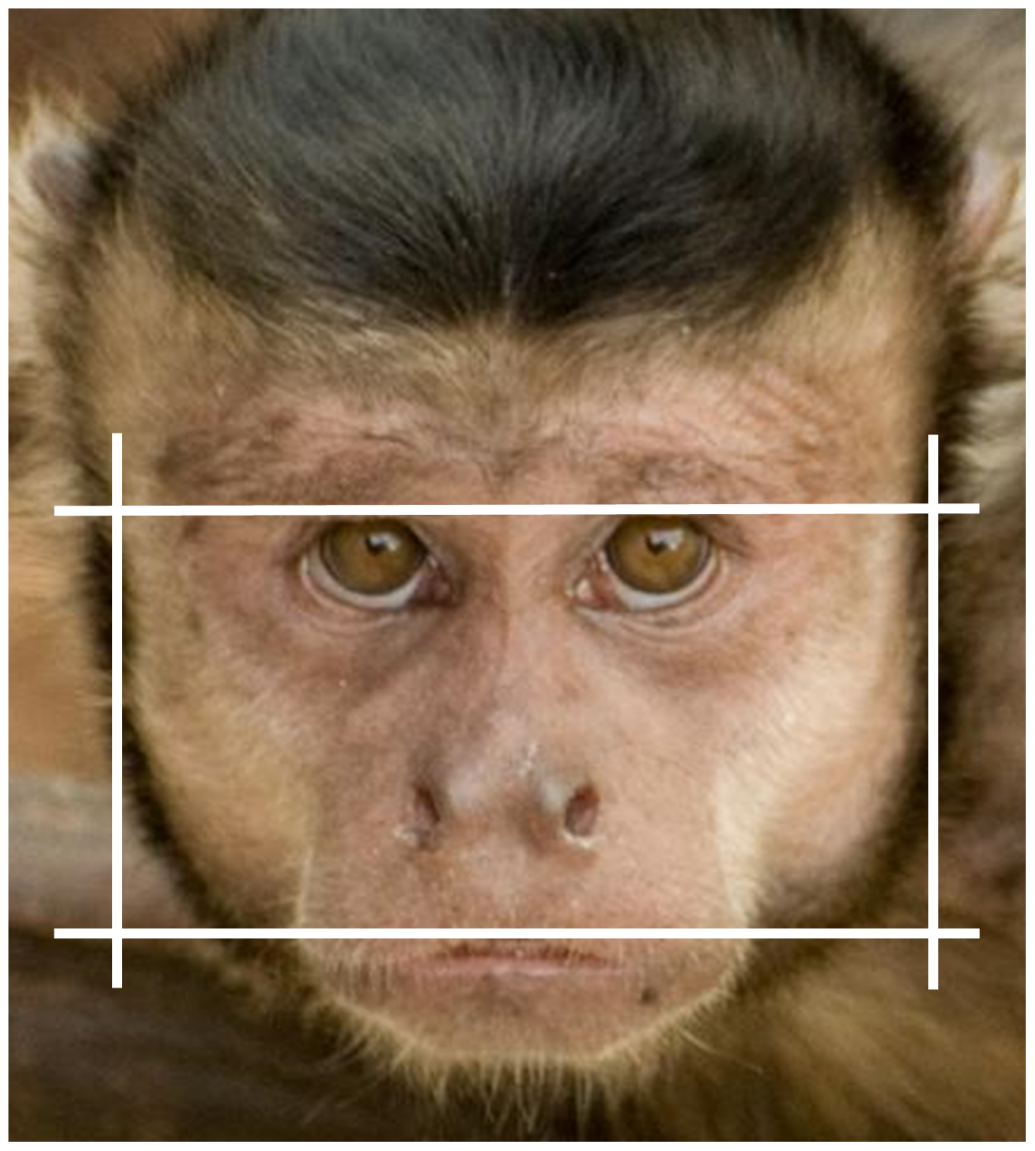
Illustration of the facial width-to-height ratio: zygomatic width (distance between vertical lines) divided by upper face height (distance between horizontal lines).

Measures were based on frontal facial photographs. Prior to measurement, photographs were aligned and scaled according to interpupillary distance. fWHR was then computed as the ratio of bizygomatic-width (maximum horizontal distance from the left to the right facial boundary) to upper face height (vertical distance from the mid-point of the upper lip to the highest point of the eyelids; see Figure 1) using Psychomorph [39]. Measurement reliability was good (ICC = .86) based on a subset of photographs (N = 18) measured twice. In addition, measures from several photographs per individual (mean = 4.69, SD = 2.44) were averaged in order to maximise the signal to noise ratio. All images were taken within 1 calendar year, thus controlling for longitudinal changes. At the time of measurement, the researcher was blind to the assertiveness levels or alpha status of the individuals that were measured.

### Alpha Status and Assertiveness Measures

#### Alpha status

Alpha status was assessed by observation of behaviours including wariness of other group members, being sought out for mating, number of offspring, frequent grooming, and ability to take food from humans and other monkeys (see [32]). In capuchins, the highest-ranking individual is recognised as having alpha status, which in addition to being dominant is also associated with several traits including assertiveness, unprovoked deference by subordinates, central position in the main party of the group and, at least in the wild, a leadership role in group-movements. The combination of these traits allow for easy and straight-forward recognition of alpha status in capuchins. Within each social group, one male and one female were accorded alpha status, yielding a total of 18 alpha individuals. The alpha-status of each individual was indicated by a number of raters, and there was complete inter-rater agreement for alpha status assignment. Raters had at least one year of experience working with the monkeys from their site. Alpha status was furthermore related to objective measures of social rank as well as Assertiveness (see next section for details).

#### Individual differences in assertive personality

Assertiveness was assessed using the Hominoid Personality Questionnaire [40]. Details of this analysis can be found in Morton et al. [33]. Briefly, 127 study subjects (64 of which were also used in the present study) were rated on 54 items by researchers and handlers familiar with the individuals being rated (X+SD = 3.24+1.61 raters). Subjects were rated on each adjective, using a 7-point scale ranging from 1 (no expression) to 7 (high expression). Each item consisted of an adjective paired with 1–3 sentences defining it within the context of primate behaviour. For instance, *fearful* was defined as “Subject reacts excessively to real or imagined threats by displaying behaviours such as screaming, grimacing, running away or other signs of anxiety or distress”. Reliability of ratings within and across raters was good (ICC+SD = .63+0.14), therefore all raw ratings were entered into a Principle Components Analysis. Five components were identified from these ratings: Assertiveness, Openness, Neuroticism, Sociability, and Attentiveness. Individual t-scores were calculated for each monkey on each of the five personality dimensions, and these scores predicted relevant behaviour up to one year later (e.g. scores on Sociability positively correlated with time each monkey spent in close proximity to others [33]). Thus, ratings were considered to be valid measures of real-world behaviour among the study subjects.

Here we use individual scores on Assertiveness as a measure of dominance-linked behaviour in our 64 subjects. The highest loadings for this dimension were bullying (.93), aggressive (.92), and dominant (.91) (see table 1 for full component structure) [33]. Assertiveness was positively correlated with behaviours typical of dominance in *Sapajus* (e.g. time spent grooming and aggressing others; [32], [33]). Assertiveness was also positively associated with alpha status, in both males (*t*_33_ = 6.69, *p*<0.001, 95% CI [1.04, 1.96]) and females (*t*_25.6_ = 5.35, *p*<0.001, 95% CI [0.90, 2.02]) indicating that this factor captured behaviour relevant to their dominance hierarchy. There was no difference between sexes for Assertiveness scores (*t*_41_ = 1.03, *p* = 0.31), suggesting relatively low sexual differentiation on this trait, but also reflecting possible rater biases towards rating individuals within sex categories. Assertiveness was validated as a measure relevant to status in a sub-sample of individuals for which social rank data were available (N = 18); Assertiveness was strongly correlated with social rank in these monkeys (r = .67, p<.01), which was calculated using data on the number of aggressive displays given/received by each individual (i.e. David’s scores; see [41]).

**Table 1:** Salient loadings of assessed personality attributes on Assertiveness, adapted from Morton and colleagues [32].

Trait	Assertiveness Component Loading
Bullying	0.92
Aggressive	0.91
Stingy/Greedy	0.88
Dominant	0.83
Jealous	0.82
Irritable	0.67
Independent	0.61
Manipulative	0.59
Reckless	0.53
Defiant	0.48
Anxious	−0.49
Fearful	−0.57
Dependent/Follower	−0.63
Cautious	−0.67
Timid	−0.68
Vulnerable	−0.75
Gentle	−0.81
Submissive	−0.89

### Statistical Analyses

Potential differences in age, sex ratio, fWHR and Assertiveness between sites were assessed using ANOVA. We tested possible sexual dimorphism in fWHR and relationships to adulthood using ANOVA. We tested the hypothesis that fWHR undergoes age-related changes focused around puberty by performing a linear regression between fWHR and age (in years). We assessed whether fWHR was predictive of alpha status and Assertiveness in adults using a logistic regression. Because we also hypothesised sexual dimorphism, sex and the interaction of sex × age were included as covariates. To test whether weight mediated sex differences in adults, a regression analysis and bootstrapping were conducted with weight as mediator, sex as a predictor and fWHR as the outcome variable. All statistical analyses were performed in R version 2.15 [42], with alpha set at 0.05, two-tailed. The raw data used for analyses can be found in the supporting information.

## Results

There were no significant differences between sites for either age (*F*_2,61_ = 1.4, *p* = .25, η_p_^2^ = .04), sex (*F*_2,61_ = 1.27, *p* = .29, η_p_^2^ = .04), fWHR (*F*_2,61_ = 0.28, *p* = .76, η_p_^2^ = .01) or Assertiveness (*F*_2,61_ = 0.23, *p* = .79, η_p_^2^ = .01). Data were therefore collapsed across the three sites.

**Figure 2:**
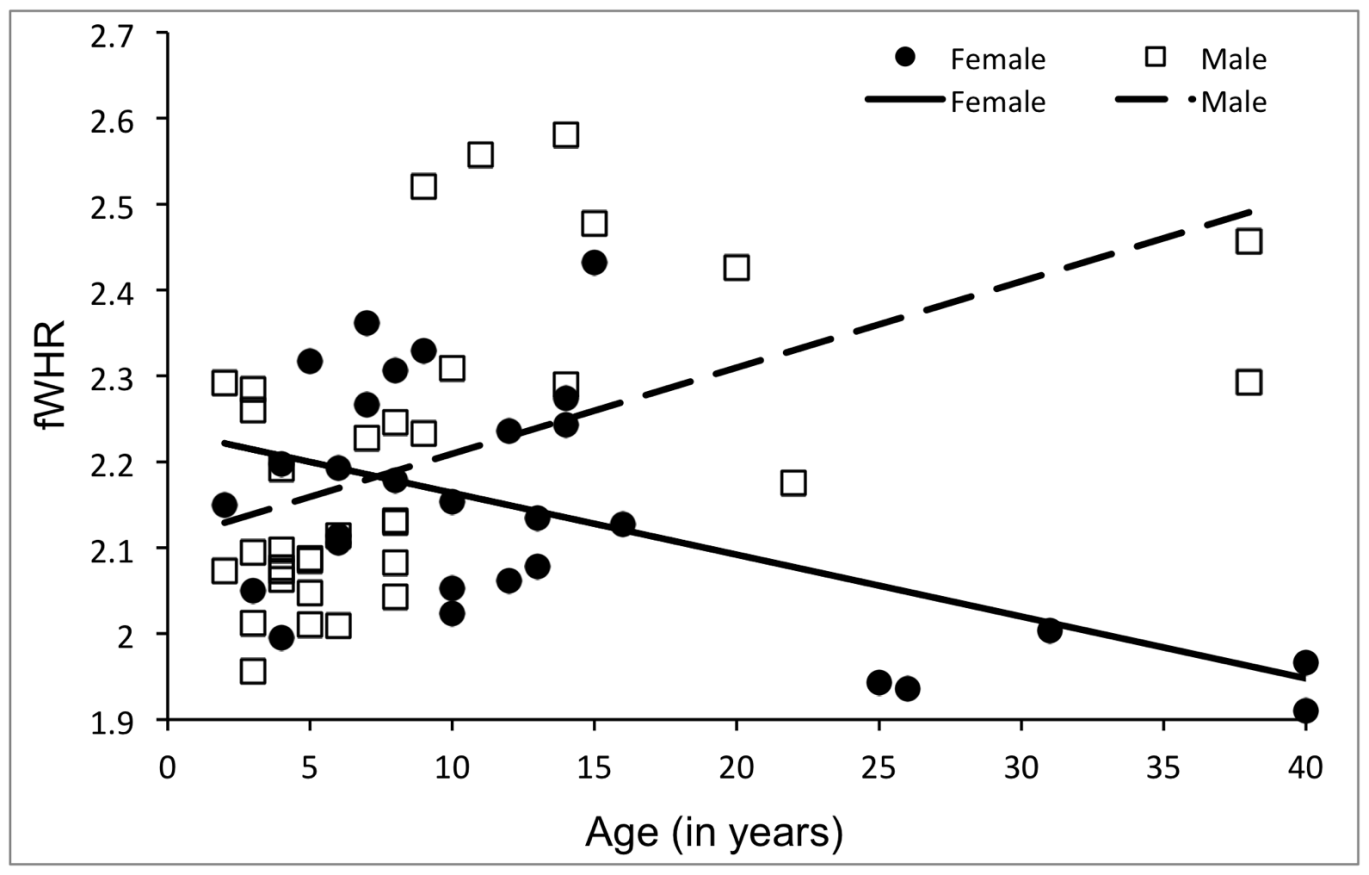
Linear effects of age and sex on fWHR. In males, fWHR increases significantly with age, suggesting developmental changes at puberty. In females, fWHR appears to decrease over the lifespan, although no significant change is observed when excluding animals older than 20 years.

The first hypotheses tested were that fWHR would be sexually dimorphic in *Sapajus* [6], and, that this dimorphism would emerge only in mature individuals following testosterone exposure at puberty. To test this, fWHR in male and female subjects was contrasted using ANOVA. There was no significant sex difference in fWHR across the whole sample (*F*_1,62_ = 2.15, *p* = .15 η_p_^2^ = .03). We next tested sex differences independently in adult and juvenile groups. Among adults, i.e. individuals who were six years or older, (*F*_1,41_ = 7.70, *p* = .008, η_p_^2^ = .16), males (*M* = 2.28 *SD* = 0.18) showed higher fWHR than females (*M* = 2.14 *SD* = 0.14). By contrast, there were no significant difference in fWHR between male (*M* = 2.11 *SD = *0.10) and female (*M* = 2.14 *SD = *0.13) juveniles (*F*_1,19_ = .427, *p* = .52, η_p_^2^ = .02). To assess whether the sex difference in adult individuals was linked to developmental changes of fWHR, we tested continuous effects of age on fWHR using regression models, entering fWHR as the dependent variable, with sex, age, and the interaction of sex × age as predictors. Both the main effect of sex (*β* = 0.38, *p* = .03) and the interaction of sex × age (*β* = −0.77, *p*<.001) were significant, while age effects did not reach significance (*β* = 0.08, *p* = .47; overall model: *F*_3,60_ = 8.13, *p*<.001, *R* ^2^ = .29; Figure 2).

To ensure that developmental status × sex effects on fWHR were related to pubertal developmental changes in fWHR and dominance rank, rather than being influenced by changes specific to old-age, the regression analysis was replicated excluding all 9 animals over 20 years of age. Age effects now reached significance (*β* = 0.50, *p*<.001) with sex × age (*β* = −0.70, *p* = .005) and sex (*β* = 0.46, *p* = .06) remaining predictors (although marginally for sex) in this reduced sample (overall model: *F*_3,51_ = 8.40, *p*<.001, *R* ^2^ = .33).

**Figure 3:**
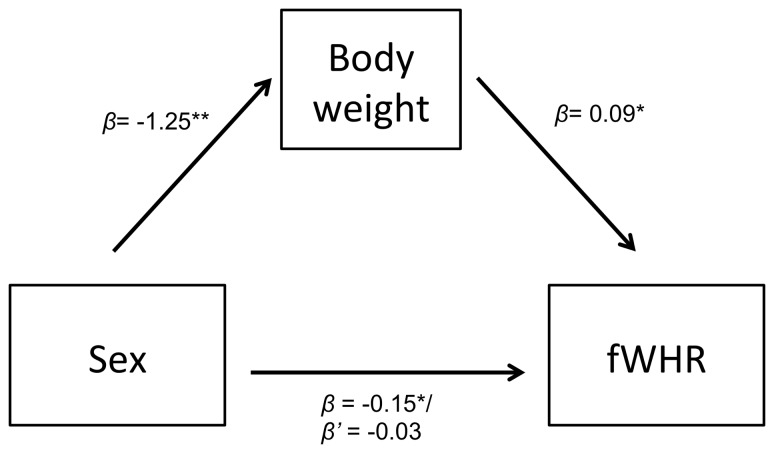
Mediation model of sex differences in fWHR by weight.

Hypothesized mediation effects of weight on sex differences in adults [20] were tested in the sub-sample of adults that had weight data available (N = 34) following Preacher and Hayes’ [43] model. The analysis showed a significant relationship (a) between sex and weight (*β* = −1.24, p<.001), (b) between weight and fWHR (*β* = 0.09, p = .002), and (c) between sex and fWHR (*β* = −0.15, p = .01); this relationship disappeared after controlling for weight (*β* = −0.03, p = .57). Bootstrapping suggested significant mediation (indirect effect = −0.12, CI[-.21,-.01]; Figure 3).

We next moved to test the relationship between fWHR and dominant behaviour, our core hypothesis. Hypothesis three predicted that fWHR would be associated with alpha status and Assertiveness. Since only adult individuals can gain alpha status, this prediction was tested in the adult sample only. An initial independent t-test revealed that alpha individuals had significantly larger fWHR compared to adult non-alpha individuals (t(41) = 3.45, p = .001). To further investigate this relationship, we next ran a logistic regression, with age and sex as control variables. In this test, fWHR (*β* = 7.86, *p* = .008) significantly predicted alpha status (overall model: *X^2^*_3_ = 15.89, *p* = .001; *Nagelkerke R^2^* = .42), controlling for sex (*β* = −0.72, *p* = .37) and age (*β* = 0.09, *p* = .04). The association further held when weight was entered as an additional control variable for the subset of individuals that had weight data available: alpha status was significantly predicted by fWHR (*β* = 7.09, *p* = .03) with no other variable reaching significance (all *p*>.09; overall model: *X^2^*_4_ = 10.93, *p* = .03).

**Figure 4:**
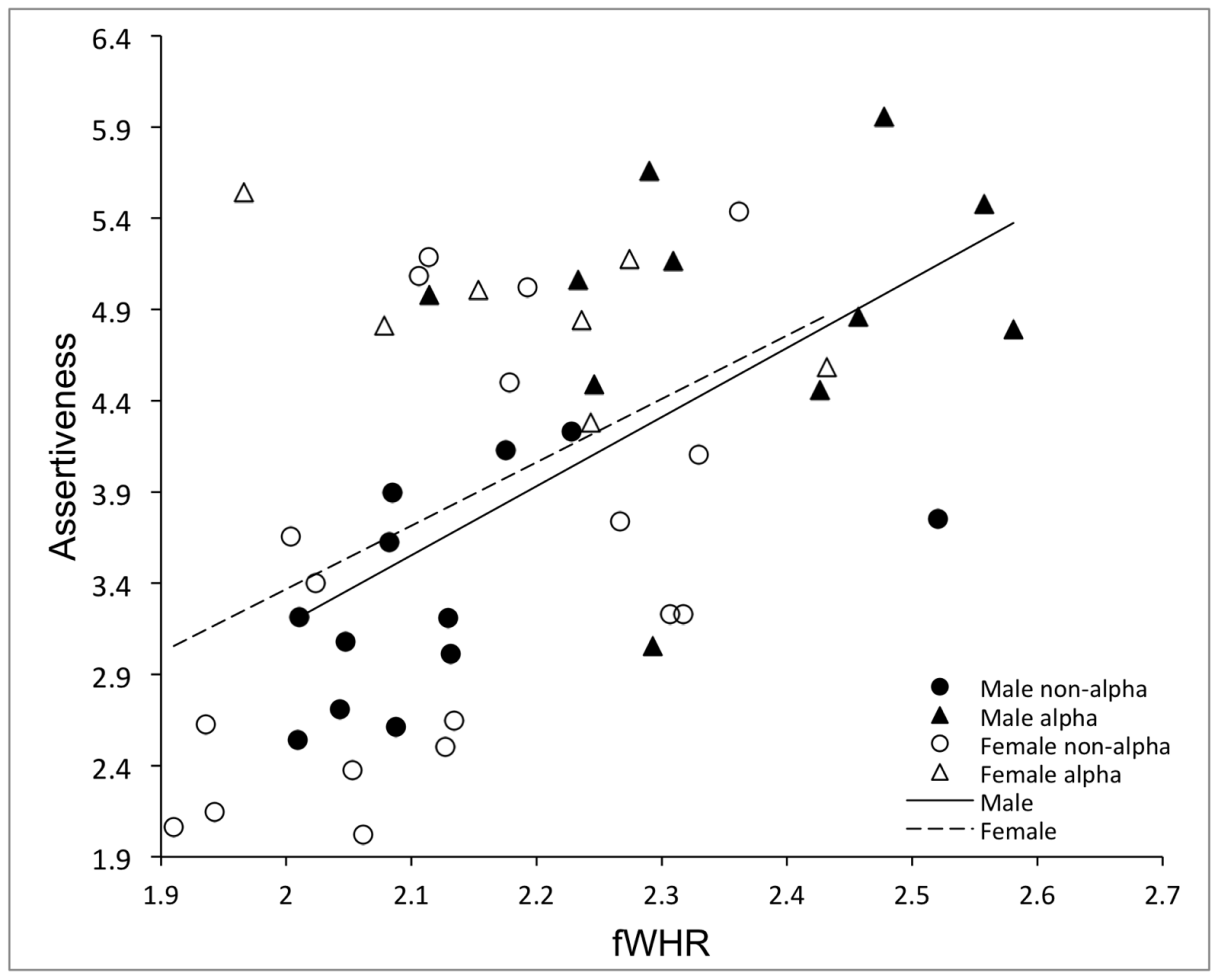
Association of Assertiveness and fWHR in adult males and females, split by alpha status. In both sexes a significant positive linear relationship between fWHR and Assertiveness is visible. This relationship held when examining non-alpha individuals only.

In order to assess whether the differences between alpha and non-alpha fWHR could be accounted for by a physiological response to gaining alpha status, we further assessed whether Assertiveness among all adult animals was predicted by fWHR when controlling sex and age (Figure 4). The overall model was significant (*F*_3,39_ = 5.49, *p* = .003, *R* ^2^ = .30). Within this, Assertiveness was significantly predicted by fWHR (*β* = 0.55, *p* = .001) but not by sex (*β* = 0.07, *p* = .66) or age (*β* = −0.07, *p* = .62). To test whether this association was exclusively driven by alpha individuals, we next assessed whether fWHR predicted Assertiveness in non-alpha adult individuals. The association between Assertiveness and fWHR remained significant following this restriction (fWHR: *β* = 0.43, *p* = .05). In the juveniles, there was no association between Assertiveness and fWHR (*β* = 0.01, *p* = .97); the overall model, with sex and age controlled, was non-significant (*F*_3,17_ = 0.39, *p* = .76, *R* ^2^ = .06). Additionally, we assessed whether in adult individuals alpha status had a moderation effect on the link between fWHR and Assertiveness. A regression model with fWHR, alpha status, fWHR × alpha status, age and sex predicting Assertiveness revealed a marginal moderation effect of alpha status among adults (*β* = 0.45, *p* = .09).

Finally, we assessed possible influences of body weight on the relationship between Assertiveness and fWHR in the sub-sample that had weight measures available. The association of weight with Assertiveness was not significant (*r* = .28, *p* = .10), and controlling for weight using a linear regression of age, sex, weight, and fWHR on Assertiveness indicated that the relationship between Assertiveness and fWHR remained significant (*β* = 0.58, *p*<.01) with effects of sex (*β* = −0.03, *p* = .88), age (*β* = 0.01, *p* = .98), and weight (*β* = −0.11, *p* = .65) being non-significant predictors of Assertiveness.

## Discussion

Our results indicated that fWHR is a sexually dimorphic trait in *Sapajus,* (although this dimorphism may be mediated by a dimorphism in body weight). In addition, *Sapajus* fWHR is closely associated with status and associated behavioural traits (i.e. assertive personality) in both adult males and adult females. This link emerged in both sexes after puberty and, unlike the evidence for sexual dimorphism, survived correction for body weight.

In capuchins, while it is possible to clearly identify and rate behavioural traits associated with dominance (e.g. aggressive wins/loses), it can be more difficult to place individuals into a precise ranking order of dominance given their relatively low rates of aggression and high levels of social tolerance compared to other primate species [32]. We therefore used Assertiveness as a measure of each monkey’s relative social dominance because this measure provides a validated trait-level assessment of the behaviour of each individual across time and contexts, where each individual can be placed along a continuous gradient ranging from high to low Assertiveness. Moreover, within the Living Links population, individual differences in social status (determined by calculating David’s scores using data on aggression given/received; see [41]) positively associated with scores on Assertiveness up to one year later. As such, our results support a specific link between facial structure and personality traits related to dominant behaviour in capuchins, irrespective of the group-level ranking of individuals. Nonetheless, future research assessing other measures of status or dominant behaviour will be valuable in order to establish cross species comparable links between behaviour and appearance. In particular, in the current study we did not use direct quantitative measures of dominance, which may limit the conclusions that can be drawn from the current data.

The relationship between alpha status/Assertiveness and fWHR in both sexes runs contrary to reports in humans where the link between dominant behaviour and fWHR has been found exclusively among males (e.g. [8], [10], [12]). One explanation for this discrepancy might be that human and *Sapajus* females show different behaviours associated with dominance. For example, while numerous studies in humans indicate that men exhibit dominant behaviour and aggression to a much larger extent than women (e.g. [44], [45]), in *Sapajus*, females are commonly observed to aggress against other females and even males, indicating perhaps that hierarchies are less sexually differentiated in *Sapajus* than in some other primate species (e.g. baboons, macaques) [32]. Thus, unlike humans, both male and female *Sapajus* may be exposed to similar selection pressures associated with dominant behaviour. While it is conceivable that the associations between face shape and behaviours linked to dominance in females reported here are specific to brown capuchins, further comparative work is necessary to test for such face-behaviour associations in a range of other primate species with varying levels of social dominance (e.g. despotic versus egalitarian species).

Weston et al. [7] previously detected sex differences in *Sapajus* fWHR using measurements taken from skulls. Here, we confirm that these sex differences exist in *Sapajus* fWHR using surface-based measurements. Importantly, this dimorphism was mediated by sex differences in body weight in the sub-sample that had weight measurements available, indicating a lack of sexual dimorphism in fWHR when size correlates are controlled, which reflects findings in humans (e.g. [20]). These results thus confirm the importance of controlling for body size when examining fWHR. To better understand the underlying mechanism(s) that link fWHR to dominant behaviour in *Sapajus* and other species, it would be of particular value to examine the sex-specificity of the behavioural correlates of fWHR (e.g. aggression), and associated endocrine profiles.

The association between fWHR and age was not significant within female *Sapajus*, suggesting that fWHR remains relatively stable throughout a female’s life span; however, additional larger studies would be valuable to confirm this finding. In contrast, male fWHR was positively associated with age, suggesting an increase during sexual maturation, with adult males having a significantly larger fWHR compared to adult females and to juveniles of both sexes. These findings may indicate that male sex hormones (such as testosterone) are involved in the development of fWHR [25].

To examine the evolution of fWHR and canine size as cues to dominance linked behaviours in primates, it will be necessary to measure the association between these physical traits and behaviours associated with dominance related traits in other primate species. The lack of a significant sex difference in human fWHR [14], [19], [20] and canine size [21] suggests that canine size – previously argued to account for lower fWHR dimorphism in species such as *Gorilla* [7] – cannot fully account for species differences in fWHR-dimorphism. In other words, fWHR is not an obligate substitute for canine dimorphism.

Our results indicate that the same facial features are linked to competitive behaviour across different species. Indeed, humans and *Sapajus* last shared a common ancestor about 43 million years ago [46]. Thus, the existence of an association between fWHR and dominance associated behaviours in both species suggests that the relationship is phylogenetically old, perhaps derived through common selective pressures associated with dominance. However, as we have noted, further data are needed on species that vary in their display of dominance (e.g. egalitarian versus despotic species) and sexual dimorphism in order to fully understand commonality of selection pressures and behaviours.

While it is currently unclear whether facial width provides an anatomical advantage over and above mere cueing of dominance linked behaviours, at least two possibilities deserve mentioning. First, fWHR may be linked to bite strength or, in other words, superior weaponry. The masseter muscle, responsible for bite force, runs below the zygomatic arch. Thus, larger muscles that afford greater bite strength may require the zygomatic arch to be positioned more laterally, hence a greater facial width. In this case, fWHR could be a cue to bite strength, which is a marker related to dominance in several species (e.g. [47]). Second, fWHR may indicate a robust skull structure. In humans, males have stronger skulls than females, perhaps to resist fracture from blows typically encountered during fights [48]. Within males, a wider zygomatic arch may relate to a stronger skull structure, thus indicating greater ability to withstand injury during fighting encounters. Future work testing these predictions would be valuable to the understanding of relationships between fWHR and behaviours linked to dominance across species. Irrespective of possible anatomical advantages however, the current data suggest that intra-sexual selection through status competition and fighting has likely shaped the primate face.

In summary, this study demonstrates an association between facial shape and dominance related behaviour in a nonhuman species. These findings suggest a phylogenetically old link between facial structures and behaviour and underline the likely importance of such links. Further research will be needed to determine whether fWHR is used by *Sapajus* as a cue for dominance linked behaviours, particularly when encountering unfamiliar individuals (e.g. dispersing males or neighbouring groups), and whether this trait is associated with advantages to the bearer (e.g. frequency and level of aggression received from others).

## Supporting Information

Data S1 **Raw data presented in this manuscript.**
(XLSX)
